# Association of cognitive impairment and breast cancer survivorship on quality of life in younger breast cancer survivors

**DOI:** 10.1007/s11764-021-01075-x

**Published:** 2021-06-26

**Authors:** Diane Von Ah, Adele D. Crouch, Patrick O. Monahan, Timothy E. Stump, Frederick W. Unverzagt, Susan Storey, Andrea A. Cohee, David Cella, Victoria L. Champion

**Affiliations:** 1 Indiana University School of Nursing, 600 Barnhill Drive, NU 120, Indianapolis, IN 46202 USA; 2 Department of Biostatistics, Indiana University School of Medicine, HS3000, Indianapolis, IN 46202 USA; 3 Department of Psychiatry, Indiana University School of Medicine, 355 W. 16th Street, Suite 2800, Indianapolis, IN 46202 USA; 4 Department of Medical Social Sciences, Northwestern University Feinberg School of Medicine, 625 N. Michigan Ave – 21st Floor, Chicago, IL 60611 USA; 5 Institute for Public Health and Medicine-Center for Patent Centered Outcomes, Northwestern University Feinberg School of Medicine, 625 N. Michigan Ave – 21st Floor, Chicago, IL 60611 USA; 6 IU Simon Cancer Center, Indiana University School of Nursing, 600 Barnhill Drive, NU 318, Indianapolis, IN 46202 USA

**Keywords:** Breast cancer survivor, Cognitive impairment, Quality of life, Physical well-being, Psychological well-being

## Abstract

**Purpose:**

Younger breast cancer survivors (BCS) often report cognitive impairment and poor quality of life (QoL), which could be interrelated. The purpose of this study was to examine the association of cognitive impairment and breast cancer status (BCS versus healthy control (HC)), with QoL, which included psychological (depressive symptoms, well-being, perceived stress, and personal growth) and physical well-being (physical functioning and fatigue).

**Methods:**

Four hundred ninety-eight BCS (≤45 years at diagnosis) who were 3 to 8 years post-chemotherapy treatment and 394 HC completed subjective questionnaires and a one-time neuropsychological assessment, including tests of attention, memory, processing speed, and verbal fluency. For each test, cognitive impairment was defined as scoring 1.5 and 2.0 standard deviations below the mean of the HC group. Separate linear regression models for each outcome were ran controlling for known covariates.

**Results:**

BCS reported significantly more memory problems than HC (*p* < 0.0001), with up to 23% having significant impairment. Cognitive performance did not differ significantly between BCS and HCs. BCS vs. HCs had greater depression and fatigue, yet more personal growth. Objective and subjective cognitive impairment were significantly related to greater depressive symptoms and perceived stress and lower well-being and physical functioning; whereas, objective impairment was related to less personal growth and subjective impairment was related to greater fatigue.

**Conclusions:**

Younger BCS report significant cognitive impairment years after treatment which may relate to greater decrements in QoL.

**Implications to Cancer Survivors:**

Assessment and interventions to address cognitive concerns may also influence QoL outcomes in younger BCS.

**Supplementary Information:**

The online version contains supplementary material available at 10.1007/s11764-021-01075-x.

## Introduction

Breast cancer survivors (BCS) make up the largest population in the cancer survivor community. With over 3.8 million BCS in the USA [1], focus on their quality of life is imperative [2]. BCS often experience decrements in quality of life across the cancer trajectory [2–5]. While some report improvement with the cessation of treatment, quality of life concerns, including poorer psychological and physical well-being, can persist long after treatment [6–9]. Younger BCS often report poorer quality of life than older BCS [3, 8, 10, 11] and express concerns regarding lingering symptoms, including cognitive impairment [12]. Cognitive impairment, commonly reported by BCS, include deficits in memory, speed of processing, attention, concentration and working memory, and language and executive functioning [13, 14]. These impairments in cognition may persist for many years post-treatment [15, 16] and have also been associated with decrements in quality of life [17–19]. However, previous studies have failed to examine whether quality of life was related to breast cancer survivorship, cognitive impairment, or both in younger BCS. Therefore, the purpose of this study was to examine the association of cognitive impairment and breast cancer status (BCS versus healthy control (HC)), with quality of life, which for this study included psychological (depressive symptoms, well-being, perceived stress, and personal growth) and physical well-being (physical functioning and fatigue). Research questions tested included: (1) Are there differences in cognitive impairment and quality of life between BCS and HC? and (2) Is cognitive impairment and breast cancer status (BCS vs. HC) associated with quality of life variables, including psychological and physical well-being?

## Methods

Data used for this study were part of a larger cross-sectional, descriptive quality of life study comparing younger BCS, older BCS, and healthy age-matched controls (HC), collected through an Eastern Cooperative Oncology Group (ECOG) 97-site database [8]. Details of the parent study and results excluding neuropsychological assessment data have been reported elsewhere [8]. Briefly, younger BCS eligible and interested were contacted by study personnel and once consented completed survey questionnaires, a neuropsychological assessment battery as well as provided the name and contact information of 3 women who were within 5 years of their age for comparison. Eligibility criteria included female BCS who were (1) diagnosed with stages I–IIIa breast cancer at ≤ 45 years of age; (2) 3 to 8 years post-treatment, which included chemotherapy; and (3) free of current/history of major medical, neurologic, or psychiatric illness. Healthy controls (no history of breast cancer) were frequency age-matched with BCS within ± 5 years. This study was approved by the Institutional Review Board.

## Measures

### Demographic and Medical Information

Sociodemographic (e.g., age, race, education, and household income) and medical information (e.g., cancer history, treatment, and cancer stage) were collected through self-report and medical record review.

### Cognitive Impairment Assessment

Standardized neuropsychological assessments [20, 21] were administrated by trained and experienced psychometricians via telephone [22, 23]. The assessment battery took 35 min to complete and included the following tests (in order of administration). Learning and Memory: Rey Auditory Verbal Learning Test (AVLT) [24, 25] a 15-item, 5-trial word list learning task in which sum recall is the total number of words recalled across all five learning trials and delayed recall is free recall of the list after completion of the remaining tests in the battery. Attention, Concentration and Working Memory: Digit Span from the WAIS-III [26] requires verbal repetition of ever longer digit strings forward and then backward. Total score is the number of strings correctly recalled. Speed of Processing: Symbol Digit Modalities Test: Oral Response Version [27] requires decoding a series of symbols by verbally stating the number that should be paired with each symbol by reference to a constantly available legend or key. Verbal Fluency: Controlled Oral Word Association (COWA) [28] is a test of verbal fluency that requires the spontaneous production of words beginning with a given letter with the total number recorded. The Squire Subjective Memory Questionnaire Scale (SSMQ) [29] has 18 items, which assess subjective memory functioning on a 9-point scale, with higher scores indicating better memory function. Cronbach alpha coefficients were 0.93 for both BCS and HC.

### Quality-of-Life Assessment

Quality of life is a multi-dimensional construct [30] and was defined by two major dimensions including psychological well-being and physical well-being. Psychological well-being was measured by four proxy variables including depressive symptoms, overall well-being, perceived stress, and positive change. Depressive symptoms: self-report of depressive symptoms was measured by the Center for Epidemiologic Studies-Depression Scale [31]. This 20-item scale uses a 4-point Likert-type response scale, with higher scores indicating greater depressive symptoms. The Cronbach alpha coefficients were 0.90 for BCS and 0.89 for HC. Overall well-being: The Index of Well-Being (IWB) [32] is a 9-item scale that measures well-being. The IWB asks participants to rate how they feel about their lives on a 7-point semantic scale, with higher scores indicating greater well-being. Cronbach alpha coefficients were 0.92 for both BCS and HC. Perceived Stress: Perceived stress was measured by the Impact of Event Scale-Revised (IES-R). The IES-R assesses for stress disorder on a 5-point Likert-type scale, with higher scores indicating higher stress. BCS rated distress regarding their breast cancer and HC subjects identified their own personal stressor within the last 12 months. The Cronbach alpha coefficients were 0.91 for both BCS and HC. Personal growth (positive change): The Post-traumatic Growth Inventory (PTGI) was used to assess perceived personal growth or positive change after trauma [33]. This 21-item Likert-type scale assesses positive change on a 6-point scale, with higher scores indicating more positive change. Internal consistency was high for both BCS with Cronbach alpha coefficients of 0.94 and 0.96 for BCS and HC, respectively.

Physical well-being was measured by two proxy variables including physical functioning and fatigue. Physical functioning was measured by the Physical Functioning Scale (PF-10) [34] . The PF10, a 10-item Likert-type scale that assesses the extent to which health limits everyday physical activities on a three-point scale from 1 (yes, limited a lot) to 3 (no, not limited at all). Higher scores reflect better physical functioning. Cronbach alpha coefficients were 0.88 for BCS and 0.91 for HC. Fatigue was measured by the Functional Assessment of Cancer Therapy-Fatigue (FACT-F) [35]. The FACT-F is a 13-item instrument in which participants rate fatigue-related items on a 5-point Likert-type scale, with lower scores indicating greater fatigue. Cronbach alpha coefficients were 0.94 for BCS and 0.93 for HC.

### Data Analysis

Data analysis was conducted using SAS version 9.4 [36]. Descriptive statistics were used to describe the major variables. General linear models, using two-sided partial *t*-tests adjusted for potentially confounding covariates (age, education, race, and income), were conducted to compare differences in BCS and HC on neuropsychological tests and self-report variables. The composite neuropsychological score was determined for each individual patient as the average of the standard *Z* scores over all five neuropsychological cognitive test scores. Significant cognitive impairment on each neuropsychological test and overall composite (across the 5 tests) was defined on a standardized *Z* score metric after adjusting for demographics by (1) regressing each cognitive score on demographics among the control group only, (2) applying this control regression equation to BCS to calculate BCS predicted values (i.e., values expected if the BCS were a control with the same demographics) and BCS residuals (predicted minus observed values), (3) standardizing by dividing residuals by the control group SD, and (4) comparing the standardized residuals for each group (BCS, control) to a cutoff of −1.5 (and −2.0 for sensitivity analysis) [37]. By definition, approximately 7% of HC will have standardized residuals less than 1.5 when data are normally distributed; thus, the important information is the extent to which BCS impairment exceeds that of HC. The *Z* score cutoffs of −1.5 SD (for mild cognitive impairment) and −2.0 SD (for mild-moderate cognitive impairment) are consistent and correspond to approaches by Tanner-Eggen and colleagues 2015 [38] and the International Cancer and Cognition Task Force (ICCTF) [39], respectively. Count and percent of impaired participants were cross-tabulated by test. General linear models were run to determine the association of each cognitive score (independent variable) and breast cancer survivorship (BCS vs. HC; independent variable) with quality of life measures (dependent variable), controlling for age, education, race and income. Each model included survivorship group, and to avoid multicollinearity, a single cognitive score. Standardized coefficients (STB) and two-sided partial *t*-test *p* values (adjusted for covariates) were reported from these models. For depressive symptoms, logistic regression was used in a sensitivity analysis based on a common clinical threshold for depression (CES-D ≥ 16). The interaction was tested between breast cancer survivorship (BCS vs. HC) and each cognitive domain on the quality of life outcomes. All tests were two sided, using significance level of 0.05 for main effects and 0.01 for interaction effects.

## Results

A total of 895 females (BCS, *n* = 498 and healthy control *n* = 397) participated in this study. BCS were on average were 45 years (*SD* = 4.8) of age at survey (ranging in age between 28 and 54). The BCS were primarily White, college educated, and were on average 6 years post-diagnosis. The HC were frequency age-matched to BCS within 5 years, yielding a similar age distribution (HC, M = 46.6, SD = 7.1, range 26–59; BCS, M = 45.3, SD = 4.8, range 28–54), which was statistically significant (*p* = .003). All of the BCS had received chemotherapy and the majority had received radiation therapy (69%) and over one-third were currently taking an anti-hormonal therapy (39.4%). Table 1 displays demographic data for BCS and HC as well as medical data for BCS.

**Table 1:** Description of the sample—BCS versus healthy control (*N* = 892)

Demographic	Total	BCS	Healthy control	*p*_1_
	*N* = 892	*N* = 498	*N* = 394	
Age at survey (self report; mean (SD))	45.9 (6.0)	45.3 (4.8)	46.5 (7.1)	0.0025**
Years of education (mean (SD))	15.0 (2.6)	14.8 (2.6)	15.1 (2.5)	0.1456
Income (*N* (%))				
<$30,000	85 (9.7)	48 (9.8)	37 (9.6)	0.0913
$30,000–75,000	335 (38.4)	172 (35.2)	163 (42.3)	
>$75,000	453 (51.9)	268 (54.9)	185 (48.1)	
Race (*N* (%))				
Caucasian	814 (91.3)	454 (91.2)	360 (91.4)	0.4288
African American	44 (4.9)	22 (4.4)	22 (5.6)	
Other	34 (3.8)	22 (4.4)	12 (3.0)	
Total number of comorbidities (mean (SD); median; range)	1.3 (1.5); 1; 0–11	1.3 (1.5); 1; 0–11	1.4 (1.6); 1; 0–8	0.2572
Stage of cancer (*N* (%))				
Stage 1		114 (22.9)	na	na
Stage 2		308 (61.9)	na	na
Stage 3		66 (13.3)	na	na
Type of surgery (*N*(%))				
Mastectomy		268 (53.8%)	na	na
Lumpectomy		230 (46.2%)	na	na
Radiation therapy given (*N* (%))		319 (69.4)	na	na
Current use of estrogen-blocking therapy (*N* (%))		195 (39.2)	na	na
Years since diagnosis (mean (SD))		5.9 (1.5)	na	na

Notes: missing values were excluded for years of education (*n* = 12), income (*n* = 19), stage (*n* = 10), and radiation therapy (*n* = 38)

*p*_*1*_, *p* value for comparison across all the two groups (chi-square used for categorical variables, two-sided *t*-test used for continuous variables)

*p < .05, **p < .01, ***p < .001

Significant findings were highlighted in bold; p-values were inserted in the table notes and * inserted with significant values

Table 2 displays the adjusted mean, standard deviation, and percent impairment for each cognitive domain, composite cognitive score, and subjective (self-report) memory. Self-rated memory function (SSMQ) for BCS was significantly below that of the HC (*p* ˂ 0.0001). There were no significant differences between the BCS and HC on objective tests of new learning and memory (AVLT sum recall and AVLT delayed), attention, concentration, and working memory (digit span), speed of processing (symbol digit), verbal fluency (COWA), or the total composite score across the five separate tests.

**Table 2:** Comparison of cognitive performance and subjective symptoms for breast cancer survivors (*n* = 498) and healthy controls (*n* = 394)

Cognitive domain, objective neuropsychological tests and subjective memory	BCS		HC		Difference (BCS-HC)		−1.5 SD		−2.0 SD	
	*N*	Adjusted mean (95% CI)	*N*	Adjusted mean (95% CI)	Adjusted mean difference (95% CI)	*p* value	% BC impaired^a^	% HC impaired^b^	% BC impaired^c^	% HC impaired^d^
Memory, sum recall, AVLT	486	49.8 (48.5, 51.1)	374	50.1 (48.8, 51.5)	−0.33 (−1.38, 0.72)	.5320	7.2	4.8	1.4	2.7
Memory, delayed recall, AVLT	485	9.8 (9.3, 10.2)	373	9.9 (9.4, 10.4)	−0.11 (-0.47, 0.25)	.5465	10.3	7.2	4.7	2.4
Attention, concentration, and working memory, digit span	485	18.7 (17.9, 19.4)	374	19.0 (18.2, 19.8)	−0.31 (−0.90, 0.27)	.2952	5.4	4.6	0.2	1.3
Speed of processing, symbol digit	485	53.6 (52.2, 55.1)	374	52.6 (51.1, 54.1)	1.01 (−0.15, 2.16)	.0870	3.1	4.0	0.6	1.3
Verbal fluency (COWA)	485	39.0 (37.2, 40.8)	374	39.6 (37.8, 41.5)	−0.61 (−2.02, 0.80)	.3959	5.6	4.6	1.0	1.1
Overall neuropsychological test composite*	485	−0.3 (−0.5, −0.1)	374	−0.2 (−0.4, −0.1)	−0.05 (−0.17, 0.08)	.4590	5.4	6.2	2.1	1.3
Self-reported memory, SSMQ	485	88.7 (85.3, 92.0)	374	100.8 (97.2, 104.3)	**−12.06 (−14.74, -9.37)**	<.0001***	22.5	5.4	11.3	1.6

Notes: Estimates were obtained from a general linear model adjusted for current age, race, years of education, and income level. *p* value indicates comparison of adjusted means for younger BCS vs. HC (two-sided partial *t*-test). Higher cognitive scores indicate better performance on objective tests (AVLT, digit span, symbol digit, COWA, composite). Higher scores indicate better memory on the SSMQ

*p < .05, **p < .01, ***p < .001

^a, b^Cognitive impairment calculated as a standardized residual (observed score minus predicted score) less than −1.5, using predicted values from a control-group demographic-adjusted equation, and dividing the residual by the control group SD

^c, d^Cognitive impairment calculated as a standardized residual (observed score minus predicted score) less than −2.0, using predicted values from a control-group demographic-adjusted equation, and dividing the residual by the control group SD

*Composite calculated by taking average score of all 5 memory variables (AVLT sum recall, AVLT delayed, digit span, symbol digit, and COWA) after *Z* score standardization (mean = 0, SD = 1) using the control group mean and SD

Significant findings were highlighted in bold; p-values were inserted in the table notes and * inserted with significant values

Significant cognitive impairment was calculated for each cognitive domain, composite score, and self-reported memory. BCS reported significantly greater memory dysfunction with 109 (22.5%) of survivors showing deficits versus 20 (5.4%) of HC using the −1.5 cutoff. Using a −2.0 cutoff, BCS reported significantly greater memory dysfunction with 55 (11.3%) of survivors showing deficits versus 6 (1.6%) of HC (see Table 2). No significant differences between BCS and HCs were noted on objective neuropsychological tests; rather, deficits in cognitive performance were noted by a small sub-sample of BCS across the five objective tests with the poorest performance noted in delayed memory (AVLT delayed recall) with 51 or 10.3% demonstrating significant impairment.

Table 3 displays the comparisons between BCS and HC on psychological and physical well-being variables. Quality of life outcomes were statistically different between the groups, for depressive symptoms, perceived stress, personal growth, physical functioning and fatigue, but not for overall well-being. BCS had significantly greater depressive symptoms (*p* < 0.0001) and fatigue (*p* < 0.0001), and worse physical functioning (*p* = 0.0209), than their HC counterparts. BCS also had statistically significant greater positive change than HC participants (*p* ˂ 0.0001). However, HC had significantly greater perceived stress than the BCS (*p* < 0.0001).

**Table 3:** Comparison of quality of life variables for breast cancer survivors (*n* = 498) and healthy controls (*n* = 394)

Outcomes	BCS		HC		*Difference (BCS–HC)*	
	*N*	Adjusted mean (95% CI)	*N*	Adjusted mean (95% CI)	Mean (95% CI)	*p* value
**Psychological well-being**						
Depressive symptoms, CES-D	486	13.4 (11.9, 14.8)	373	10.7 (9.2, 12.3)	2.64 (1.46, 3.82)	<.0001***
Life satisfaction and well-being, IWB	484	11.5 (11.1, 11.9)	374	11.4 (11.0, 11.8)	0.13 (−0.17, 0.42)	.3880
Perceived stress, IES-R	485	16.2 (14.1, 18.3)	371	19.5 (17.3, 21.7)	−3.32 (−5.01, −1.64)	.0001**
Personal growth/positive change, PTGI	487	73.3 (69.5, 77.2)	371	58.8 (54.8, 62.8)	14.56 (11.49, 17.64)	<.0001***
**Physical well-being**						
Physical function, PF10	487	81.0 (78.0, 84.0)	374	83.8 (80.7, 86.9)	−2.83 (−5.23, −0.43)	**.0209**
Fatigue, FACT-F	487	37.1 (35.4, 38.7)	374	39.4 (37.7, 41.2)	−2.39 (−3.71, −1.06)	.0004**

Notes: Estimates were obtained from a general linear model adjusted for current age, race, years of education, and income level. *p* value indicates comparison of adjusted means for younger BCS vs. HC (two-sided partial *t*-test). Higher scores indicate more depressive symptoms, greater satisfaction and well-being, more stress, greater personal growth, better physical functioning, and less fatigue

*p < .05, **p < .01, ***p < .001

Significant findings were highlighted in bold; p-values were inserted in the table notes and * inserted with significant values

Table 4 displays the general linear model results for comparing the primary cognitive tests (neuropsychological cognitive composite; subjective memory) with quality of life, controlling for known covariates including age, education, race, and household income. Supplementary Table 1 shows these same results for each of the individual neuropsychological tests. Table 4 contains standardized coefficients from each separate model that includes one quality-of-life outcome (dependent variable) and two primary predictors of interest (a cognitive impairment test score and group (BCS vs HC). The models in Table 4 included main effects only. It was determined in a separate set of models with interaction terms that *none* of the relationships between cognitive performance and quality of life outcomes were modified by breast cancer status (BCS vs HC; i.e., non-significant interaction with cognitive performance), *except* for self-report memory and fatigue, as described below. The following results summarize the findings for Table 4 and Supplementary Table 1 by each quality of life outcome. In general, the results for the individual neuropsychological tests were consistent with those for the neuropsychological composite.

**Table 4:** Standardized coefficients of cognitive impairment test scores and group (BC and HC) with Quality of Life, including psychological and physical well-being

Predictors	Outcomes (dependent variables)					
	Psychological well-being				Physical well-being	
	Depressive symptoms	Life satisfaction and well-being	Perceived stress	Personal growth (positive change)	Physical functioning	Fatigue
Cognitive composite	−0.11**	0.08*	−0.13***	−0.07*	0.13***	0.04
BCS vs. HC	0.14***	0.03	−0.13***	0.30***	−0.07*	−0.12***
Squire subjective memory (SSMQ)	−0.29***	0.15***	−0.12***	0.06	0.17***	0.28***
BCS vs. HC	0.06	0.07*	−0.17***	0.32***	−0.03	−0.04

Note. Values in table cells are standardized coefficients obtained from a general linear model adjusted for current age, race, years of education, and income level. Each cell represents results from a separate linear regression model. Higher cognitive scores indicate better performance on composite test; higher scores indicate better memory on the SSMQ. Higher scores indicate more depressive symptoms, greater satisfaction and well-being, more stress, greater personal growth, better physical functioning, and less fatigue

*p < 0.05; **p < 0.01; ***p < 0.001

Significant findings were highlighted in bold; p-values were inserted in the table notes and * inserted with significant values

### Depressive Symptoms

Memory (AVLT sum recall, *p* < 0.01 and ALVT delayed, *p* < 0.05), attention, concentration, and working memory (Digit span), (*p* < 0.001), total cognitive composite (*p* < 0.01), and subjective memory (SSQM) (*p* < 0.001), as well as group status (BCS, *p* < 0.05 − < 0.001) was significantly related to depressive symptoms. Poorer cognitive performance on these individual tests, the overall cognitive composite and survivorship status (BCS vs. HC) was related to greater depression. Speed of processing (symbol digit) and verbal fluency (COWA) were not related to depressive symptoms, whereas BCS (*p* < 0.001) had significantly greater depressive symptoms than HC in those models. Results were similar in a sensitivity analysis where logistic regression was used to model clinical depression (CES-D ≥ 16) as the dependent variable.

### Well-being

Attention, concentration and working memory (digit span) (*p* < 0.001) and total cognitive composite (*p* < 0.05) were significantly related to well-being, with better cognitive performance related to greater sense of well-being. Self-reported memory (SSQM) (*p* < 0.001) and BCS status (*p* < 0.05) was significantly related to well-being, with poorer memory and being a BCS related to poorer well-being. Memory (AVLT sum recall and AVLT delayed recall), speed of processing (symbol digit), and verbal fluency (COWA) were not related to well-being.

### Perceived stress

Memory (AVLT sum recall, (*p* < 0.05), AVLT delayed recall, (*p* < 0.01), attention, concentration, and working memory (digit span), (*p* < 0.05), speed of processing (symbol digit) (*p* < 0.05), total cognitive composite (*p* < 0.001), and subjective memory (SSMQ) (*p* < 0.001) and HC (*p* < 0.001) were related to stress. Poorer cognitive performance and being a HC participant was associated with significantly greater stress. Verbal fluency (COWA) was not related to perceived stress.

### Personal growth/positive change

Memory (AVLT sum recall, *p* < .05) total composite (*p* < .05), and BCS (*p* < .01 - *p* < .001) were related to positive change. Better cognitive performance and being a BCS was related to greater positive change. Memory (AVLT delayed recall), attention, concentration, working memory (Digit Span), speed of processing (Symbol digit), verbal fluency (COWA), and subjective memory were not significantly related to positive change.

### Physical function

Memory (AVLT sum recall, (*p* < .05), AVLT delayed recall, *(p* < .05), attention, concentration, working memory (Digit Span) *(p* < .05), speed of processing (Symbol Digit) *(p* < .01), total cognitive composite (*p* < .001), and subjective memory (SSQM) (*p* < .001) and being HC *(p* < .05) was related to physical function. Better cognitive scores, self-reported memory and being a HC participant was related to better physical functioning. Verbal fluency (COWA) was not related to physical functioning.

### Fatigue

Self-reported memory (SSQM) (*p* < .001), but not BCS status (p = 0.188), was significantly related to fatigue, with poorer subjective memory related to greater fatigue. However, there was a significant interaction (p = 0.002) between subjective memory and BCS status on fatigue. The relationship between subjective memory and fatigue was stronger for BCS (STB = 0.34, p < 0.001) than HC (STB = 0.12, P = 0.028).

BCS status was significantly related to fatigue in each model *(p* < .001). However, performance on objective tests including memory (sum recall and delayed memory), attention, concentration, working memory, speed of processing, verbal fluency, and total cognitive composite were not related to fatigue.

## Discussion

Almost half of all BCS are younger than 45 years of age at diagnosis [1]. These younger BCS often report poorer quality of life than HC or older BCS counterparts [3, 8, 40]. Additionally, researchers have noted that quality of life for younger BCS tends to worsen overtime [11]. Many studies have also identified that younger survivors report more symptoms post-treatment [3], but few have focused on cognitive concerns. This study was one of the first to our knowledge to tease out the relationship between cognitive impairment (both subjective report and objective neuropsychological assessments) and BCS status and their relationship with quality of life outcomes (psychological and physical well-being) in younger BCS. Previous work by Amidi and colleagues (2015) focused on a sub-sample of older BCS (64-75 years of age) and noted no differences in subjective cognitive impairment from normative data [41]. However, we found that younger BCS reported significantly poorer memory when compared to age-matched HC participants. Almost one-quarter of the younger BCS (22%) expressed significant memory concerns compared to just 5% of HC using the -1.5 standardized demographic-adjusted residual cutoff. These results are similar to a recent study by Gregorowitsch et al. [12] who assessed subjective cognitive function in 715 BCS and noted that younger BCS had more pronounced subjective cognitive impairment compared to older BCS up to 24 months post-treatment. Our study extends these findings to younger BCS who were on average 6 years (range 3–8 years) post-treatment and suggests reports of cognitive impairment may linger for younger BCS. Our impairment rates were 11% for BCS compared to 2% for HC when using the −2.0 cutoff, indicating a substantial number of younger BCS incurs mild-moderate cognitive impairment.

Although BCS reported significantly more cognitive concerns, there was not a significant difference noted on any of the objective neuropsychological tests or the cognitive composite score. These results differ from previous studies in all-aged BCS [42] and older BCS (≥60) compared to HC [43, 44]. Instead, we noted that there was only a small subset of younger BCS (3.1–10.3%) with significant cognitive impairment with the largest difference noted in delayed memory (10.3%). The failure to find significant differences in objective cognitive impairment may in part be due to the methods employed in this study. The cross-sectional nature does not allow for the identification of intra-individual variability over time [45]. Longitudinal research, including cognitive performance pre-chemotherapy would allow for a more complete assessment of cognitive impairment in younger BCS. In addition, the use of multiple tests assessing the same cognitive domain would increase reliability of assessing the domain versus performance on one standardized test [46]. Researchers have also identified other factors such as older age [13, 47, 48] and poorer cognitive reserve (capacity) [13, 47, 48] and other comorbidities (cardiotoxicity) [13] may be important risk factors for developing cognitive impairment after cancer and cancer treatment and warrant further investigation [13].

### Quality of life - psychological well-being

The relationship between psychological well-being and BCS status and cognitive impairment varied depending on the outcome measure utilized. Younger BCS had significantly higher levels of depressive symptoms than HC. Similarly Maass et al. [49], in a comparison study of 350 BCS to 350 HC, found that the odds of depression and severe depression were greater in BCS than age-matched HC, even after adjusting for history of depression or prescription of antidepressant use. Taken together, younger BCS appear at greater risk for depressive symptoms and depression than HC long after cancer and cancer treatment and should be routinely assessed throughout the cancer care trajectory as an integral part of the survivorship care plan [50].

Breast cancer status was related to personal growth, but not perceived stress. HC participants reported greater current perceived stress than BCS; but this may have been due to differences in the timeframe and variance in type of stressors identified with the IES. However, BCS did report greater personal growth or positive change compared to HC, which often happens through and after the occurrence of a stressful life event, such as a cancer diagnosis. Our results are similar to previous findings in cancer survivors who have found a greater appreciation for life after cancer diagnosis and treatment [51, 52].

Perceived stress measured by the IES was negatively related to both objective and subjective cognitive impairment. This finding is consistent with a study by Hermelink and colleagues (2017) who found that post-traumatic distress mediated the relationship between breast cancer and cognitive performance (Go/NoGo test) [53]. The nature of this relationship between psychological stress and cognitive impairment needs further exploration.

Cognitive function was significantly associated with psychological well-being. Although, significance varied depending on the specific cognitive domains and the specific psychological well-being outcomes. The overall cognitive composite (summary of all objective tests) was significantly related to depressive symptoms, overall well-being, perceived stress, and personal growth. These findings underscore the significant association that cognitive impairment may have on the psychological well-being of younger BCS.

Objective (all domains except speed of processing) and subjective memory impairment was significantly related to greater levels of depressive symptoms in these younger BCS. Although subjective cognitive impairment has been consistently associated with depression in BCS [54], findings with objective cognitive impairment have been mixed. Only two studies have noted this relationship between objective measures of attention [55] and executive function [56] and depression in BCS. This may be due to the fact that most studies examining cognitive impairment exclude survivors with a history of or current depression [57]. Thus, further research is needed to fully understand this important relationship between cognitive impairment and depression in younger BCS overtime.

### Quality of life - physical well-being

BCS status was significantly related to physical well-being. Younger BCS had significantly poorer physical functioning and greater fatigue than HC comparators. BCS often report fatigue as a common and debilitating symptom, even years after treatment [58]. Fatigue may interfere with BCS ability to participate in meaningful life activities, including social activities and work. In fact, researchers have noted that the greater fatigue severity, the greater the interference with work ability in cancer survivors [59]. Similarly, poor physical functioning has also been linked to negative outcomes, including failure to return to work or poor work ability in BCS [60]. This is especially important to younger BCS, who often identify returning and engaging in meaningful work as a sign of full recovery [61]. More work is needed to aid younger BCS to maintain their physical functioning and promote positive long-term outcomes.

Objective cognitive impairment was also highly correlated with worse physical functioning, but not with fatigue, in these younger BCS. Physical functioning and activity have been linked with cognitive impairment in BCS. Hartman et al. [62] found associations between greater physical activity and better cognitive performance in 136 early stage BCS. Interventional research targeted to improve physical functioning/activity should be explored for their beneficial effects on cognitive performance in BCS [63].

We also found that subjective memory impairment was significantly related to both physical functioning and fatigue. Additionally, a striking interaction result showed that the relationship between greater subjective memory impairment and greater fatigue was even stronger for BCS than it was for HC. Fatigue and subjective cognitive impairment have been shown to be highly correlated in BCS [64]. Perceived cognitive impairment and its relationship to physical functioning and fatigue are important because, beyond being an indicator of quality of life, fatigue and physical functioning have been shown to predict longer recurrence-free and overall survival [65] and mortality in BCS, respectively [66].

## Limitations

Findings should be considered in light of the limitations of the study. The cross-sectional study design limited the findings to associations and no causal inferences can be drawn. Additionally, more work is needed regarding how to more accurately assess stress to be a reliable comparison to HC participants. And finally, more specific treatment-related data (type and dose of chemotherapy, etc.) would assist in future studies in directly tying the type of treatment to those at greatest risk for cognitive impairment.

## Conclusions and implications for cancer survivors

Younger BCS in this study reported significant subjective cognitive impairment that is still prominent 3 to 8 years post-treatment. These findings have implications for quality survivorship care. The healthcare team needs to ensure that they are assessing younger BCS for cognitive impairment across the cancer survivor trajectory. Cognitive assessments should pre-date adjuvant therapy and be incorporated into cancer survivorship care planning. In addition, as recommended in the NCCN guidelines [67], clinicians should be assessing for and treating psychological distress, depressive symptoms, and other correlated symptoms which may also impact cognitive functioning.

Overall, our findings also suggest that objective and subjective cognitive impairment are related to a number quality-of-life outcomes, and decrements in these outcomes were found to be more strongly correlated in these younger BCS. Although more longitudinal research is needed to examine the trajectory and patterns of these relationships overtime, interventions aimed at improving cognition in younger BCS may have broader implications and impact both psychological and physical well-being.

## Supplementary Information

ESM 1 (DOCX 17 kb)

## References

[1] Miller KD (2019). Cancer treatment and survivorship statistics, 2019. CA Cancer J Clin.

[2] Ganz PA (2004). Quality of life at the end of primary treatment of breast cancer: first results from the moving beyond cancer randomized trial. J Natl Cancer Inst.

[3] Howard-Anderson J (2012). Quality of life, fertility concerns, and behavioral health outcomes in younger breast cancer survivors: a systematic review. J Natl Cancer Inst.

[4] Hoyer BB (2011). A nurse-led telephone session and quality of life after radiotherapy among women with breast cancer: a randomized trial. Open Nurs J.

[5] Cappiello M (2007). Breast cancer survivors: information and support after treatment. Clin Nurs Res.

[6] Ferreira AR (2019). Differential impact of endocrine therapy and chemotherapy on quality of life of breast cancer survivors: a prospective patient-reported outcomes analysis. Ann Oncol.

[7] Ganz PA (2000). Quality of life across the continuum of breast cancer care. Breast J.

[8] Champion VL (2014). Comparison of younger and older breast cancer survivors and age-matched controls on specific and overall quality of life domains. Cancer.

[9] Costa-Requena G, Rodriguez A, Fernandez-Ortega P (2013). Longitudinal assessment of distress and quality of life in the early stages of breast cancer treatment. Scand J Caring Sci.

[10] Kornblith AB (2007). Long-term psychosocial adjustment of older vs younger survivors of breast and endometrial cancer. Psychooncology.

[11] Bloom JR (2012). Quality of life of younger breast cancer survivors: persistence of problems and sense of well-being. Psychooncology.

[12] Gregorowitsch ML (2019). The effect of chemotherapy on subjective cognitive function in younger early-stage breast cancer survivors treated with chemotherapy compared to older patients. Breast Cancer Res Treat.

[13] Ahles TA, Root JC, Ryan EL (2012). Cancer- and cancer treatment-associated cognitive change: an update on the state of the science. J Clin Oncol.

[14] Bernstein LJ, et al. Cognitive impairment in breast cancer survivors treated with chemotherapy depends on control group type and cognitive domains assessed: a multilevel meta-analysis. Neurosci Biobehav Rev. 2017;83:417–28. 10.1016/j.neubiorev.2017.10.028. 10.1016/j.neubiorev.2017.10.028 29092778

[15] Koppelmans V (2012). Neuropsychological performance in survivors of breast cancer more than 20 years after adjuvant chemotherapy. J Clin Oncol.

[16] Stanton AL, Rowland JH, Ganz PA (2015). Life after diagnosis and treatment of cancer in adulthood: Contributions from psychosocial oncology research. Am Psychol.

[17] Von Ah D (2013). Impact of perceived cognitive impairment in breast cancer survivors. Eur J Oncol Nurs.

[18] Reid-Arndt SA, Hsieh C, Perry MC (2010). Neuropsychological functioning and quality of life during the first year after completing chemotherapy for breast cancer. Psychooncology.

[19] Pullens MJ, De Vries J, Roukema JA (2010). Subjective cognitive dysfunction in breast cancer patients: a systematic review. Psychooncology.

[20] Lezak MD. Neuropsychological assessment. 2nd ed. New York: Oxford University Press; 1995.

[21] Golden, C.J., Espe-Pfeifer, P. & J. Wachsler-Felder, Neuropsychological Interpretations of Objective Psychological Tests (Critical Issues in Neuropsychology), 2000, Hingham: Kluwer Academic Publishers. pages 254

[22] Unverzagt FW (2007). The Indiana University telephone-based assessment of neuropsychological status: a new method for large scale neuropsychological assessment. J Int Neuropsychol Soc.

[23] Marceaux JC (2019). Verbal fluency in a national sample: telephone administration methods. Int J Geriatr Psychiatry.

[24] Lezak MD, Howieson DB, Loring DW (2004). Neuropsycholgical assessment.

[25] Rey A (1941). L'examen psychologique dans les cas d'encephalopathie traumatique. Arch Psychol.

[26] Wechsler D (1997). WAIS-III: Administration and scoring manual: Wechsler adult intelligence scale-third edition.

[27] Smith A (1982). Symbol Digit Modalities Test.

[28] Benton AL, Hamsher KD Multilingual aphasia examination. Iowa City: AJA Associates; 1989

[29] Squire LR, Wetzel CD, Slater PC (1979). Memory complaint after electroconvulsive therapy: assessment with a new self-rating instrument. Biol Psychiatry.

[30] Ferrell BR (1997). Quality of life in breast cancer survivors as identified by focus groups. Psychooncology.

[31] Radloff LS (1977). The CES-D scale: A self-report depression scale for research in the general population. Appl Psychol Meas.

[32] Campbell A, Converse PE, Rodgers WL. The quality of American life: perceptions, evolutions, and statisfactions. New York: Russell Sage Foundation; 1976.

[33] Tedeschi RG, Calhoun LG (1996). The Posttraumatic Growth Inventory: measuring the positive legacy of trauma. J Trauma Stress.

[34] McHorney CA (1992). The validity and relative precision of MOS short- and long-form health status scales and Dartmouth COOP charts. Results from the Medical Outcomes Study. Med Care.

[35] Yellen SB (1997). Measuring fatigue and other anemia-related symptoms with the Functional Assessment of Cancer Therapy (FACT) measurement system. J Pain Symptom Manag.

[36] SAS Institute, I., What's new SAS@ 9.4. 2017, SAS Institute, Inc: Cary, N.C.

[37] Rao SM (1991). Cognitive dysfunction in multiple sclerosis. II. Impact on employment and social functioning. Neurology.

[38] Tanner-Eggen C (2015). The neuropsychological assessment of cognitive deficits considering measures of performance variability. Arch Clin Neuropsychol.

[39] Wefel JS (2011). International Cognition and Cancer Task Force recommendations to harmonise studies of cognitive function in patients with cancer. Lancet Oncol.

[40] Sammarco A (2009). Quality of life of breast cancer survivors: a comparative study of age cohorts. Cancer Nurs.

[41] Amidi A (2015). Long-term subjective cognitive functioning following adjuvant systemic treatment: 7-9 years follow-up of a nationwide cohort of women treated for primary breast cancer. Br J Cancer.

[42] Von Ah D (2009). Cognitive function in breast cancer survivors compared to healthy age- and education-matched women. Clin Neuropsychol.

[43] Mandelblatt JS, et al. Cancer-related cognitive outcomes among older breast cancer survivors in the thinking and living with cancer study. J Clin Oncol. 2018:JCO1800140. 10.1200/JCO.18.00140. 10.1200/JCO.18.00140 PMC7237199 30281396

[44] Yamada TH (2010). Neuropsychological outcomes of older breast cancer survivors: cognitive features ten or more years after chemotherapy. J Neuropsychiatry Clin Neurosci.

[45] Collins B, Widmann G, Tasca GA (2018). Effectiveness of intraindividual variability in detecting subtle cognitive performance deficits in breast cancer patients - erratum. J Int Neuropsychol Soc.

[46] Yao C, Bernstein LJ, Rich JB (2017). Executive functioning impairment in women treated with chemotherapy for breast cancer: a systematic review. Breast Cancer Res Treat.

[47] Ahles TA (2010). Longitudinal assessment of cognitive changes associated with adjuvant treatment for breast cancer: impact of age and cognitive reserve. J Clin Oncol.

[48] Ahles TA. Brain vulnerability to chemotherapy toxicities. Psychooncology. 2012;21(11):1141–8. 10.1002/pon.3196. 10.1002/pon.3196 PMC3788849 23023994

[49] Maass S, et al. Long-term psychological distress in breast cancer survivors and their matched controls: a cross-sectional study. Maturitas. 2019;130:6–12. 10.1016/j.maturitas.2019.09.003. 10.1016/j.maturitas.2019.09.003 31706438

[50] Dietrich L (2016). Effectiveness of a survivorship program: an assessment of patients with breast cancer in a community setting. J Oncol Pract.

[51] Cordova MJ, Andrykowski MA (2003). Responses to cancer diagnosis and treatment: posttraumatic stress and posttraumatic growth. Semin Clin Neuropsychiatry.

[52] Cordova MJ (2001). Posttraumatic growth following breast cancer: a controlled comparison study. Health Psychol.

[53] Hermelink K, et al. Chemotherapy and post-traumatic stress in the causation of cognitive dysfunction in breast cancer patients. J Natl Cancer Inst. 2017:109(10). 10.1093/jnci/djx057. 10.1093/jnci/djx057 28521364

[54] Bedillion MF, Ansell EB, Thomas GA (2019). Cancer treatment effects on cognition and depression: The moderating role of physical activity. Breast.

[55] Freeman JR, Broshek DK (2002). Assessing cognitive dysfunction in breast cancer: what are the tools?. Clin Breast Cancer.

[56] Vearncombe KJ (2009). Predictors of cognitive decline after chemotherapy in breast cancer patients. J Int Neuropsychol Soc.

[57] Ono M (2015). A meta-analysis of cognitive impairment and decline associated with adjuvant chemotherapy in women with breast cancer. Front Oncol.

[58] Bower JE (2014). Cancer-related fatigue--mechanisms, risk factors, and treatments. Nat Rev Clin Oncol.

[59] Behringer K (2016). Cancer-related fatigue in patients with and survivors of Hodgkin lymphoma: the impact on treatment outcome and social reintegration. J Clin Oncol.

[60] Bijker R (2018). Functional impairments and work-related outcomes in breast cancer survivors: a systematic review. J Occup Rehabil.

[61] Peteet JR (2000). Cancer and the meaning of work. Gen Hosp Psychiatry.

[62] Hartman SJ (2015). Lifestyle factors associated with cognitive functioning in breast cancer survivors. Psychooncology.

[63] Campbell KL (2019). Exercise guidelines for cancer survivors: consensus statement from international multidisciplinary roundtable. Med Sci Sports Exerc.

[64] Tometich DB (2019). Pretreatment psychoneurological symptoms and their association with longitudinal cognitive function and quality of life in older breast cancer survivors. J Pain Symptom Manag.

[65] Groenvold M (2007). Psychological distress and fatigue predicted recurrence and survival in primary breast cancer patients. Breast Cancer Res Treat.

[66] Sehl M (2013). Decline in physical functioning in first 2 years after breast cancer diagnosis predicts 10-year survival in older women. J Cancer Surviv.

[67] Denlinger CS (2016). NCCN guidelines insights: survivorship, version 1.2016. J Natl Compr Cancer Netw.

