# Supplementary material for: Association of cognitive impairment and breast cancer survivorship on quality of life in younger breast cancer survivors
Source: J Cancer Surviv. 2021 Jun 26;16(4):812–22. doi: 10.1007/s11764-021-01075-x (PMC9300496; doi:10.1007/s11764-021-01075-x)
Supplement: Supplementary file 1 — (DOCX 17 kb) [file 11764_2021_1075_MOESM1_ESM.docx]

Supplemental Table 1. Standardized coefficients of cognitive impairment test scores and group (BC and HC) with Quality of Life, including psychological and physical well-being.

|  | Outcomes (Dependent Variables) | | | | | |
| --- | --- | --- | --- | --- | --- | --- |
|  | Psychological Well-being | | | | Physical Well-being | |
| Predictors | Depressive  Symptoms | Life satisfaction & Well-being | Perceived stress | Personal growth (Positive change) | Physical functioning | Fatigue |
| AVLT Sum Recall ^a^ | -0.10** | 0.06 | -0.09* | -0.08* | 0.09* | 0.05 |
| BCS vs HC | 0.14*** | 0.03 | -0.13*** | 0.30*** | -0.07* | -0.12*** |
| AVLT Delayed Recall | -0.08* | 0.04 | -0.09** | -0.04 | 0.08* | 0.04 |
| BCS vs HC | 0.14*** | 0.03 | -0.13*** | 0.30*** | -0.07* | -0.12*** |
| Digit Span | -0.11*** | 0.11*** | -0.07* | -0.01 | 0.07* | 0.05 |
| BCS vs HC | 0.14*** | 0.03 | -0.13*** | 0.30*** | -0.07* | -0.12*** |
| Symbol Digit | -0.04 | 0.02 | -0.09* | -0.07 | 0.11** | 0.03 |
| BCS vs HC | 0.15*** | 0.03 | -0.13*** | 0.31*** | -0.08* | -0.12*** |
| COWA | -0.01 | 0.03 | -0.05 | -0.03 | 0.04 | -0.03 |
| BCS vs HC | 0.14*** | 0.03 | -0.13*** | 0.30*** | -0.07* | -0.12*** |

Note. Values in table cells are standardized coefficients obtained from a general linear model adjusted for current age, race, years of education and income level. Each cell represents results from a separate linear regression model.

Higher cognitive scores indicate better performance on objective tests (AVLT, digit span, symbol digit, COWA). Higher scores indicate more depressive symptoms, greater satisfaction and well-being, more stress, greater personal growth, better physical functioning and less fatigue.

*p < .05, **p < .01, ***p < .001.
